# Transfection of interleukin-8 increases angiogenesis and tumorigenesis of human gastric carcinoma cells in nude mice

**DOI:** 10.1038/sj.bjc.6690742

**Published:** 1999-10

**Authors:** Y Kitadai, Y Takahashi, K Haruma, K Naka, K Sumii, H Yokozaki, W Yasui, N Mukaida, Y Ohmoto, G Kajiyama, I J Fidler, E Tahara

**Affiliations:** First Department of Internal Medicine, First Department of Pathology, Hiroshima University School of Medicine, Hiroshima, 734, Japan; Departments ofSurgery, Molecular Oncology, Cancer Research Institute, Kanazawa University, Kanazawa, Japan; Cellular Technology Institute, Otsuka Pharmaceutical Co. Ltd, Tokushima, 771-01, Japan; Department of Cancer Biology, The University of Texas M. D. Anderson Cancer Center, Houston, TX, 77030, USA

**Keywords:** interleukin-8, angiogenesis, gastric carcinoma, transfection

## Abstract

The growth and spread of tumour cells depends on adequate vasculature. We have previously reported that the expression of interleukin-8 (IL-8) directly correlates with the vascularity of human gastric carcinomas. To provide evidence for a causal role of IL-8 in angiogenesis and tumorigenicity of human gastric cancer, we used the lipofectin method to stably transfect the human TMK-1 gastric carcinoma cells (low endogenous IL-8) with an IL-8 expression vector or control vector. Transfection with IL-8 did not affect the proliferation of cultured cells, yet the culture supernatants of the transfected (but not control) cells stimulated proliferation of human umbilical vein endothelial cells. The IL-8-transfected and control cells were injected into the gastric wall of nude mice. IL-8-transfected cells produced rapidly growing, highly vascular neoplasms as compared to control cells. These results provide direct evidence for the role of IL-8 in the angiogenesis and tumorigenicity of human gastric carcinomas. © 1999 Cancer Research Campaign


British Journal of Cancer (1999) **81**(4), 647–653
© 1999 Cancer Research Campaign
Article no. bjoc.1999.0742

### Transfection of interleukin-8 increases angiogenesis and tumorigenesis of human gastric carcinoma cells in nude mice

**Y Kitadai[1,2], Y Takahashi[3], K Haruma[1], K Naka[2], K Sumii[1], H Yokozaki[2], W Yasui[2], N Mukaida[4], Y Ohmoto[5], G Kajiyama[1], IJ Fidler[6] and E Tahara[2]**

[1]First Department of Internal Medicine and [2]First Department of Pathology, Hiroshima University School of Medicine, Hiroshima, 734, Japan: Departments of [3]Surgery and [4]Molecular Oncology, Cancer Research Institute, Kanazawa University, Kanazawa, Japan; [5]Cellular Technology Institute, Otsuka Pharmaceutical Co. Ltd, Tokushima, 771-01, Japan; [6]Department of Cancer Biology, The University of Texas M. D. Anderson Cancer Center, Houston, TX 77030 USA

**Summary** The growth and spread of tumour cells depends on adequate vasculature. We have previously reported that the expression of interleukin-8 (IL-8) directly correlates with the vascularity of human gastric carcinomas. To provide evidence for a causal role of IL-8 in angiogenesis and tumorigenicity of human gastric cancer, we used the lipofectin method to stably transfect the human TMK-1 gastric carcinoma cells (low endogenous IL-8) with an IL-8 expression vector or control vector. Transfection with IL-8 did not affect the proliferation of cultured cells, yet the culture supernatants of the transfected (but not control) cells stimulated proliferation of human umbilical vein endothelial cells. The IL-8-transfected and control cells were injected into the gastric wall of nude mice. IL-8-transfected cells produced rapidly growing, highly vascular neoplasms as compared to control cells. These results provide direct evidence for the role of IL-8 in the angiogenesis and tumorigenicity of human gastric carcinomas. © 1999 Cancer Research Campaign

**Keywords**: interleukin-8; angiogenesis; gastric carcinoma; transfection

Angiogenesis, which is essential for tumour growth and metastasis, depends on the production of angiogenic factors by tumour cells and normal cells (Folkman, 1986). Increased vascularity enhances the growth of primary neoplasms and provides an avenue for haematogenous metastasis (Folkman, 1990). Indeed, recent studies have shown that the incidence of metastasis can be correlated with the number and density of blood vessels in breast (Weidner et al, 1991), lung (Macchiarini et al, 1992), prostate (Weidner et al, 1993), cervical (Smith-MacCune et al, 1994), oesophageal (Kitadai et al, 1998*a*), colon (Takahashi et al, 1995) and gastric carcinomas (Takahashi et al, 1996; Tanigawa et al, 1996) and melanoma (Graham et al, 1994).

There have been many studies attempting to isolate the molecular mediators of this process. Among the possibilities is interleukin-8 (IL-8), a member of the CXC chemokine family. This cytokine was initially shown to selectively stimulate chemotactic activity for neutrophils and lymphocytes (Matsushima et al, 1988, 1992). More recent studies revealed that IL-8 is multifunctional: it can induce angiogenesis (Koch et al, 1992; Strieter et al, 1992), haptotactic migration (Wang et al, 1990) and proliferation of keratinocytes and melanoma cells (Krueger et al, 1990; Schandendorf et al, 1993). Human recombinant IL-8 can induce proliferation and migration of human umbilical vein endothelial cells (HUVECs) and is potently angiogenic when implanted in the rat cornea (Strieter et al, 1992). Furthermore, IL-8 is a known angiogenic factor for human lung carcinoma (Strieter et al, 1995), human bladder carcinoma (Tachibana et al, 1997) and human prostate carcinoma (Green et al, 1997). The CXC chemokines IL-8 and interferon-α-inducible protein (IP-10) have recently been shown to regulate angiogenic activity in idiopathic pulmonary fibrosis (Keane et al, 1997).

The expression of a wide variety of growth factors/receptors and cytokines, including epidermal growth factor (EGF) transforming growth factor (TGF-α), EGF receptor (EGF-R) (Tahara, 1990, 1993), IL-1 (Ito et al, 1993) and IL-6 (Ito et al, 1997) by human gastric carcinoma cells has been shown to correlate with malignant potential. Gastric carcinoma cells also produce angiogenic factors, including basic fibroblast growth factor (bFGF) (Tanimoto et al, 1991) and vascular endothelial growth factor (VEGF) (Yamamoto et al, 1998). We have recently found that surgical specimens of human gastric carcinomas overexpress IL-8 as compared to corresponding normal mucosa, and that the IL-8 mRNA level directly correlated with the vascularity of the tumours (Kitadai et al, 1998*b*).

There have not, however, been any studies that established a causal role for IL-8 in gastric cancer angiogenesis. The purpose of this study was, therefore, to provide evidence for the causal role of IL-8 in the growth and vascularization of human gastric cancer. We show that stably IL-8-transfected human gastric cancer cells produce angiogenic activity in culture. Subsequent to orthotopic implantation into nude mice, the cells produce rapidly growing and highly vascularized tumours.

*Received 6 January 1999*
*Revised 13 April 1999*
*Accepted 22 April 1999*

*Correspondence to:* Y Kitadai

### MATERIALS AND METHODS

### Cell cultures and tumour tissues

A human gastric carcinoma cell line, TMK-1 was established in our laboratory from a poorly differentiated adenocarcinoma (Ochiai et al, 1985). HUVECs (umbilical cord, human endothelial cells) were obtained from Dainippon Pharmaceutical Co. Ltd (Osaka, Japan). TMK-1 cells were routinely maintained in RPMI-1640 (Nissui Co. Ltd, Tokyo, Japan) with 10% fetal bovine serum (FBS) (M A Bioproducts, Inc., Walkersville, MD, USA). HUVECs were maintained in gelatin-coated dishes (Falcon Laboratories, McLean, VA, USA) in MCDB 131 medium (Life Technologies, Rockville, MD, USA) containing 10% FBS and 10 ng $ml^{-1}$ bFGF (Dainippon).

### Expression vectors

A full-length IL-8 cDNA (*Eco*RI-*Eco*RI, 1.5 kb) (Matsushima et al, 1988) was inserted into the *Eco*RI site of pUC 19. A *Sma*I-*Bam*HI fragment was cloned into the *Sma*I-*Bam*HI sites of Bluescript KS(+) (Stratagene, La Jolla, CA, USA). The resultant Bluescript KS(+) was digested with *Xho*I-*Not*I and cloned into the *Xho*I-*Not*I site of the BCMGS-Neo expression vector (kindly provided by Dr H Karasuyama, Tokyo Metropolitan Research Institute of Clinical Medicine, Tokyo, Japan). Expression of IL-8 cDNA was under the control of the cytomegalovirus promoter.

### Transfection assays and production of stable cell lines

Transfections were performed by the lipofection method (Life Technologies) with the following modifications: approximately $10^6$ TMK-1 cells were plated into culture dishes (90-mm diameter) 1 day prior to transfection. On the following day, the growth medium was replaced, and 3 h later, liposomes containing 5 μg of the expression plasmid were applied to the cells and left for 6 h. After that, cell monolayers were rinsed with RPMI containing 400 μg of G418 $ml^{-1}$ (selection medium). The selection medium was changed every 3 days.

### RNA preparation and Northern blot analysis

Polyadenylated mRNA was extracted from gastric carcinoma cells using the FastTrackTM mRNA isolation kit (Invitrogen Co., San Diego, CA, USA). mRNA was electrophoresed on 1% denaturing formaldehyde/agarose gel, electrotransferred at 0.6 A to a GeneScreen nylon membrane (DuPont Co., Boston, MA, USA), and UV cross-linked with 120 000 mJ $cm^{-2}$ using a UV Stratalinker 1800 (Stratagene). Hybridizations were performed as described previously (Radinsky, 1987). Nylon filters were washed at 65°C with 30 mM sodium chloride, 3 mM sodium citrate (pH 7.2), and 0.1% sodium dodecyl sulphate (w/v).

### DNA probes

The cDNA probes used in these analyses were a 1.3-kb *Pst*I cDNA fragment corresponding to rat glyceraldehyde 3-phosphate dehydrogenase (GAPDH) (Clontech, Palo Alto, CA, USA) and a 0.5-kb *EcoRI cDNA* frgament corresponding to human *IL-8* (Matsushima et al, 1988). A 0.7 kb human VEGF cDNA probe and a 0.8-kb bFGF cDNA probe were kindly provided by Dr M Shibuya (University of Tokyo, Tokyo, Japan) and Dr J A Abraham (California Biotechnology, Inc., CA, USA), respectively. Each cDNA fragment was purified by agarose gel electrophoresis, recovered using GeneClean (BIO 101, Inc., La Jolla, CA, USA), and radiolabelled using the random primer technique with $^{32}$P-labelled deoxyribonucleotide triphosphates (Feinberg and Vogelstein, 1983).

### Enzyme-linked immunosorbent assay for IL-8 protein

IL-8 levels in cell-free culture supernatants from transfected TMK-1 cells and homogenates of xenografts were assayed by enzyme-linked immunosorbent assay (ELISA) as described previously (Kita et al, 1992; Kitadai et al, 1998*b*) using an IL-8 monoclonal antibody. The detection limit of the assay was 20 μg $ml^{-1}$, and inter- and intra-assay variations were 4.1–4.5% and 7.9–9.3%, respectively.

### Immunohistochemical staining

Consecutive 4-μm sections were cut from each study block. Sections were immunostained for IL-8 and CD31 (specific for mouse endothelial cells). Immunohistochemistry (IHC) was performed by the immunoperoxidase technique with minor modification (Gutman et al, 1995; Kitadai et al, 1998*b*). Antibodies used were a rabbit polyclonal antibody (Otsuka Pharmaceutical Co. Ltd, Tokushima, Japan) at a 1:100 dilution for IL-8, and a rat monoclonal antibody (Pharmingen, San Diego, CA, USA) for CD31. Negative controls were done using non-specific IgG as the primary antibody.

### Vessel density

Vessel count was assessed by light microscopy in IHC-stained areas of the tumour containing the highest numbers of capillaries and small venules at the invasive edge (Weidner et al, 1991). Highly vascular areas were first identified by scanning tumour sections at low power (×40 and ×100). Vessel count was determined in six such areas at ×400 field (×40 objective and ×10 ocular), and the average count of the six fields was determined. Vessel lumen was not necessary for a structure to be defined as a vessel (Weidner et al, 1991).

### Cell growth in vitro

Cells ($5 \times 10^3$) were seeded on 24-well plates (Falcon Laboratories, McLean, VA, USA) and cultured in RPMI medium in the absence or presence of FBS. The medium was changed every 48 h. Cell number was determined in triplicate cultures.

### MTT assay

HUVECs ($5 \times 10^3$) were plated into multiple 38-$mm^2$ wells of 96-well gelatin-coated plates (Falcon Laboratories, McLean, VA, USA) in cultured medium from the transfected TMK-1 cells. The cells were cultured for 2 days, when their proliferation was determined by an MTT assay (Fan et al, 1990). Fifty microlitres of MTT (40 μg $ml^{-1}$) was added to each well, incubated for 1 h, aspirated and dissolved in dimethyl sulphoxide. The intensity of colour adduct formation was measured using an ELISA plate reader. The percentage increase in cell growth was calculated as: growth stimulation (%) = $(B–A)/A \times 100$ where A is the A540 of the control cultures and B is the A540 of test cultures. IL-8 neutralization

© 1999 Cancer Research Campaign

**Table 1** Secretion of IL-8 protein by TMK-1 cells transfected with the IL-8 gene

| Cell line | Secretion of IL-8 protein (pg ml$^{-1}$ 10$^{-4}$) |
|---|---|
| TMK-1 parental | 40.5 ± 1.3 |
| TMK-1 neo | 37.4 ± 2.3 |
| TMK-1 IL-8-C1 | 42.5 ± 3.2 |
| TMK-1 IL-8-C3 | 43.2 ± 2.8 |
| TMK-1 IL-8-C9 | 754.0 ± 8.5 |
| TMK-1 IL-8-C15 | 3512.0 ± 31.0 |

Cells were incubated in medium supplemented with 10% FBS. Culture supernatants were collected after 48 h and assayed for IL-8 by ELISA as described in the Material and Methods section. The values are the mean ± s.d. of triplicate cultures. This is one representative experiment of three.

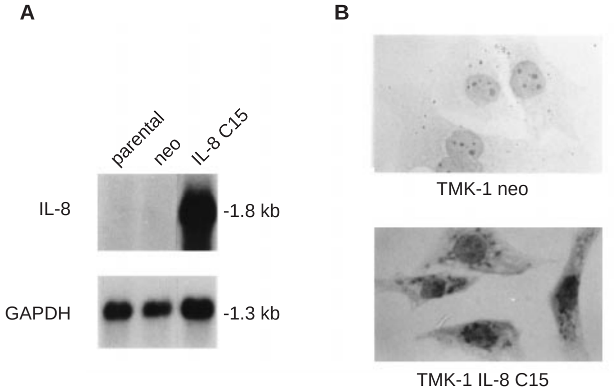

**Figure 1** Expression of IL-8 mRNA (**A**) and protein (**B**) by TMK-1 cells transfected with IL-8 gene. (**A**) Five micrograms of polyadenylated selected RNA were subjected to Northern blot analysis by using $^{32}$P-labelled IL-8 cDNA as described in Materials and Methods. A GAPDH probe was used as an internal control. (**B**) Immunohistochemistry for IL-8 of transfected TMK-1 cells. Note that IL-8 immunoreactivity is detected within the cytoplasm of TMK-1-IL-8-C15 cells but not within the TMK-1 neo cells studies used a polyclonal rabbit anti-human IL-8 antibody (Otsuka Pharmaceutical Co. Ltd).

### Growth of transfected tumour cells in nude mice

Male athymic BALB/c nude mice were obtained from Charlsriver Co. Ltd (Tokyo, Japan). The mice were maintained under specific pathogen-free conditions and used when 8 weeks old. To produce tumours, cultured cells were harvested by a brief trypsinization, and $5 \times 10^5$ or $1 \times 10^6$ viable cells were implanted into the gastric wall of nude mice as described previously (Takahashi et al, 1995). Six weeks later, the mice were killed and the tumours growing in the stomach were removed, weighed and examined histologically.

### Statistical analysis

The significance of the in vitro data was analysed by the Student's *t*-test (two-tailed), and the in vivo data was analysed by the Mann–Whitney *U*-test.

### RESULTS

### Transfection of TMK-1 cells with the IL-8 expression vector

We used the TMK-1 cell line derived from poorly differentiated adenocarcinoma because its expression level of IL-8 is extremely low (Kitadai et al, 1998*b*). First, we transfected the IL-8 expression vector (IL-8-BCMGS-neo) into the TMK-1 gastric carcinoma cells and selected stable IL-8-overexpressing clones. ELISA demonstrated that clone 15 secreted the highest level of IL-8 protein into the culture medium (Table 1); this clone was used in subsequent experiments. The IL-8 mRNA expression and cytosol localization of IL-8 protein were confirmed by Northern blot and IHC analyses respectively (Figure 1). As a control, we used the

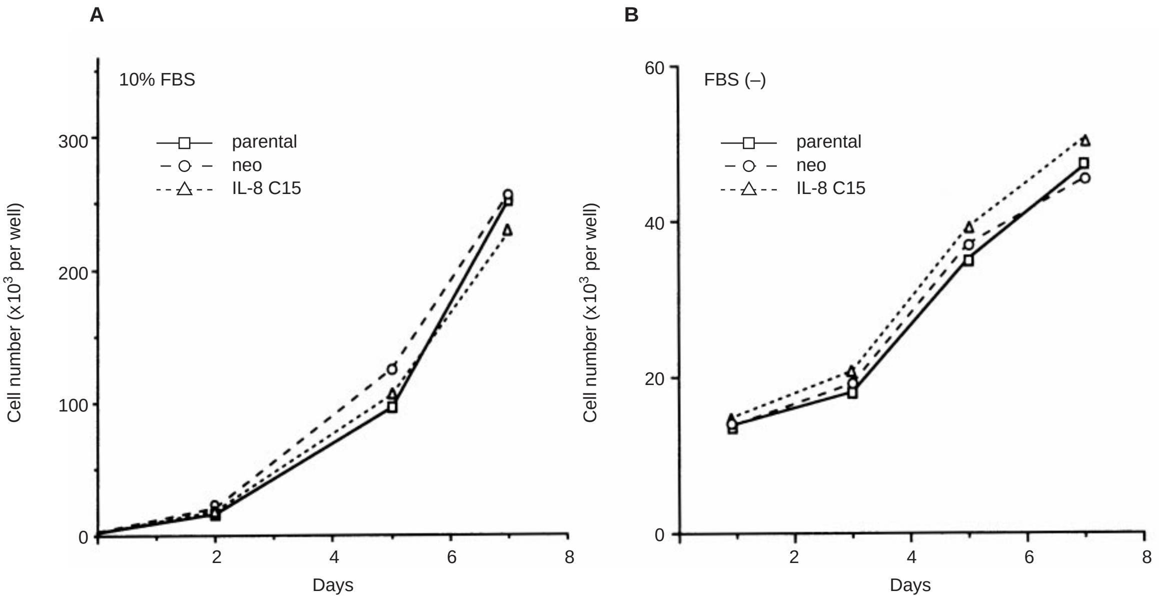

**Figure 2** In vitro growth of the TMK-1 cells transfected with IL-8 gene. Cells ($5 \times 10^3$ per well) were seeded onto 24-well plates and cultured in RPMI medium in the presence (**A**) or absence (**B**) of 10% FBS. Cell number was counted in triplicate cultures. This is one representative experiment of three

**Table 2** Growth stimulation of HUVEC by medium conditioned by IL-8-transfected TMK-1 cells

| Cell line | Growth stimulation of HUVEC cells[a] (% of control) |
|---|---|
| TMK-1 parental (control) | 100 ± 9.1 |
| TMK-1 neo | 105 ± 11.4 |
| TMK-1 IL-8-C15 | 185 ± 20.4[b] |
| TMK-1 IL-8-C15 + IL-8 Ab | 109 ± 15.6 |

[a]HUVEC cells (5 × 10$^3$ cells/well) were incubated with culture supernatants (200 μl) of TMK-1 lines. After 48 h, growth stimulation was determined by the MTT assay as described in Material and Methods. The percentage of growth stimulation was calculated by comparison with HUVEC cells cultured in medium conditioned by TMK-1 parent cells. Values are mean ± s.d. of triplicate cultures. [b]$P < 0.01$

**Table 3** Effect of IL-8 transfection on tumorigenicity of TMK-1 human gastric carcinoma cells

| Inoculum dose | Cell line | Tumorigenicity[a] | Tumour weight (mg)[b] |
|---|---|---|---|
| 5 × 10$^5$ | TMK-1 neo | 0/10 | Not determined |
| | TMK-1 IL-8-C15 | 7/10[c] | Not determined |
| 1 × 10$^6$ | TMK-1 neo | 3/10 | 12.3 ± 3.1 |
| | TMK-1 IL-8-C15 | 8/10[c] | 125.6 ± 33.4[c] |

[a]Number of positive mice/number of injected mice. [b]Mean ± s.e.m. 6 weeks after orthotopic implantation. [c]$P < 0.01$.

BCMGS-neo plasmid containing the neo-resistance gene but not IL-8 cDNA under control of the same promoter. The IL-8 production by control vector transfectant (TMK-1 neo) was similar to that of parent cells (Table 1 and Figure 1). We next examined whether the IL-8 protein produced by the IL-8 transfectant was biologically active. Since IL-8 has been reported to stimulate proliferation and migration of HUVECs (Koch et al, 1992; Kitadai et al, 1998*b*), we determined whether culture supernatants induced proliferation of HUVECs. Proliferation of HUVECs was significantly stimulated by the addition of medium conditioned by the IL-8 transfectants as compared to that from control cells. The growth stimulatory activity was blocked by neutralizing antibodies to IL-8 (Table 2).

### Effect of IL-8 transfection on in vitro growth of gastric carcinoma cells

Since IL-8 has been reported to be an autocrine growth factor for melanoma cells (Schandendorf et al, 1993), we next analysed whether IL-8 transfection stimulates the in vitro growth of gastric carcinoma cells. Under both serum-free and serum-containing conditions, cell growth was not affected by transfection with the IL-8 gene (Figure 2). This finding agreed with our previous data showing that the addition of exogenous IL-8 did not alter cell proliferation of gastric carcinoma cell lines (Kitadai et al, 1998*b*).

### In vivo growth of IL-8-transfected gastric carcinoma cells

Next, we injected the control and IL-8-transfected cells into nude mice. Since the organ microenvironment influences tumour growth and metastasis, the cells were injected into an orthotopic site (gastric mucosa) of nude mice (Fidler, 1990). As shown in Table 3, transfection of TMK-1 with IL-8 increased their tumorigenicity; namely, by 6 weeks after injection of 5 × 10$^5$ cells, IL-8-transfected cells formed tumours in seven of ten nude mice, whereas control cells (TMK-1 neo) did not form any. In addition, the size of gastric tumours produced by the 1 × 10$^6$ IL-8-transfected cells was significantly larger than that produced by control cells (Table 3). Stable expression of IL-8 mRNA and protein in the tumour lesions were confirmed by Northern blot analysis and ELISA respectively (Figure 3). Transfection with IL-8 gene did not change the expression levels of mRNA for VEGF and bFGF (Figure 3A). Immunohistochemical analysis confirmed the expression of IL-8 at the cell level. IL-8 immunoreactivity within the tumours localized mainly to cancer cells (Figure 4A). Normal epithelial cells and stromal cells showed minimal IL-8 staining with IHC (data not shown). Distant haematogeneous or peritoneal dissemination was not found in any of the injected mice.

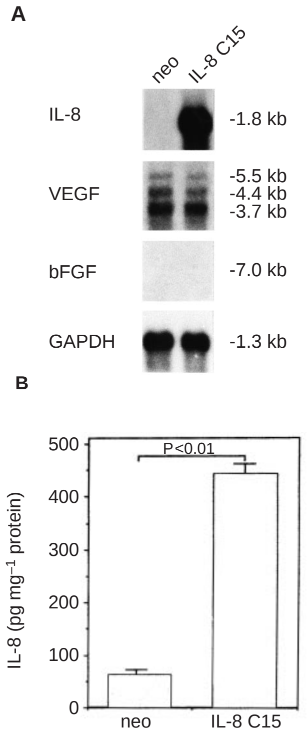

**Figure 3** Expression of IL-8 mRNA (**A**) and protein (**B**) in the TMK-1 tumours growing in the stomach wall of nude mice. (**A**) Five μg of polyadenylated selected RNA were subjected to Northern blot analysis by using $^{32}$P-labelled IL-8, VEGF, bFGF, GAPDH cDNA as described in Materials and Methods. A GAPDH probe was used as an internal control. (**B**) Tumour tissues were homogenized in PBS. Supernatants, obtained by centrifugation (1800 rpm for 10 min), were assayed for the presence of IL-8 by ELISA. The values are the mean ± s.e.m. of triplicate samples

### Effect of IL-8 transfection on angiogenesis of orthotopic xenografts

To determine whether the increased tumorigencity and growth of the tumours was associated with increased angiogenesis, we performed IHC against CD31 (mouse endothelial cell-specific) and counted microvessels in the orthotopic tumours. A representative IHC for CD31 is shown in Figure 4. The IL-8 transfection resulted in stimulation of angiogenic responses. Namely, tumour vessel density in the tumours produced by the IL-8 transfectants

 © 1999 Cancer Research Campaign

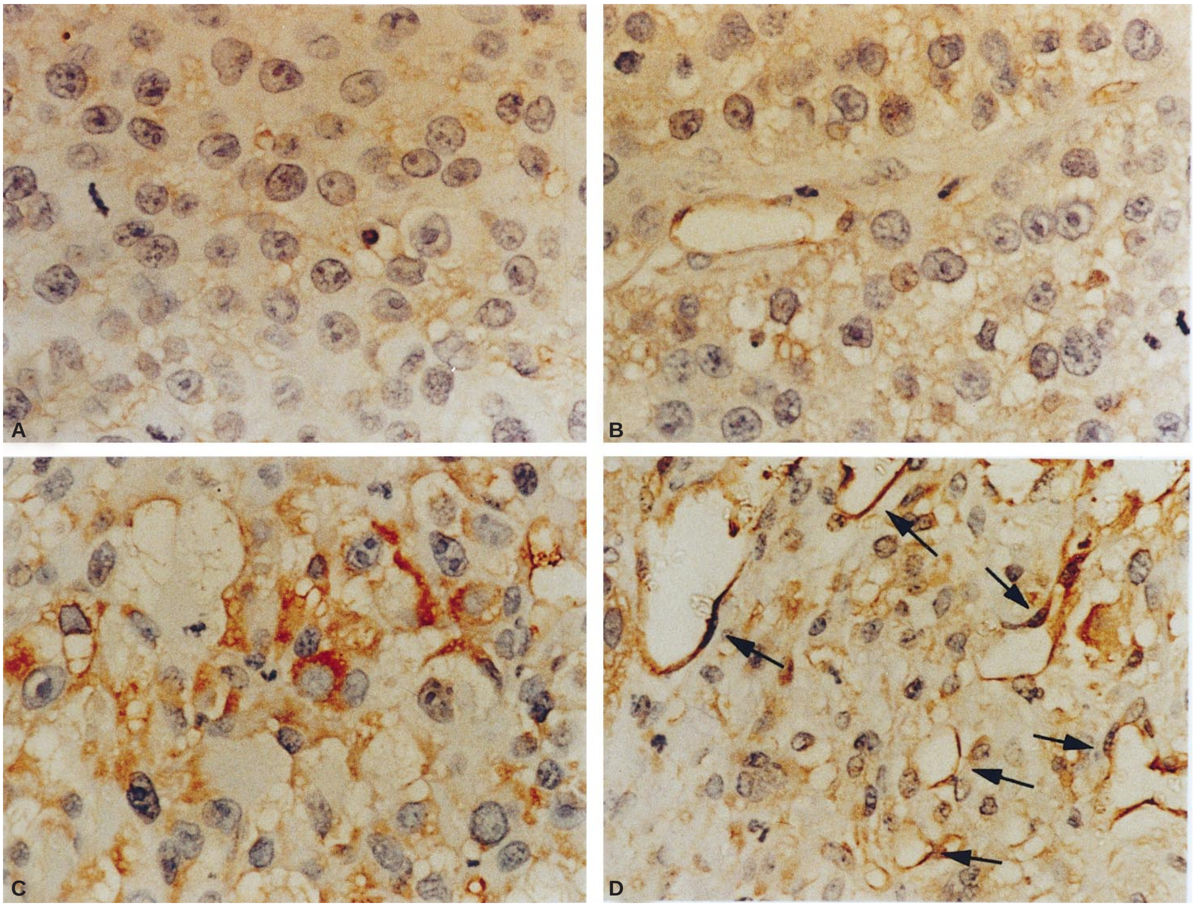

**Figure 4** Immunohistochemistry for IL-8 (**A, C**) and CD31 (**B, D**) of TMK-1 orthotopic xenografts. Note that IL-8 immunoreactivity is detected within TMK-1 IL-8-C15 cells (**C**) but not TMK-1 neo cells (**A**). The vascularity is higher in TMK-1-IL-8-C15 tumours (**D**) as compared with TMK-1 neo tumours (**B**); ×250

was significantly higher than that in control tumours (65.3 ± 5.3 ($n$ = 8) vs 21.7 ± 3.4 ($n$ = 3) vessels per HPF, $P < 0.01$).

### DISCUSSION

IL-8 is a multifunctional cytokine originally identified as a leucocyte chemoattractant (Matsushima et al, 1988, 1992). It can induce migration in some tumour cells (Wang et al, 1990) and has been implicated in the induction of angiogenesis in such diverse diseases as psoriasis (Schroeder and Christopher, 1986), rheumatoid arthritis (de Marco et al, 1991), idiopathic pulmonary fibrosis (Keane et al, 1997) and some malignant diseases (Folkman, 1986, 1990). Previous studies have demonstrated that IL-8 is expressed by lung (Strieter et al, 1995), bladder (Tachibana et al, 1997), prostate (Green et al, 1997) and head and neck squamous carcinomas (Richards et al, 1997), astrocytoma (Van Meir et al, 1992) and malignant melanoma (Schandendorf et al, 1993; Singh et al, 1994). It has been reported that IL-8 acts as an angiogenic factor in lung carcinoma (Smith et al, 1994; Strieter et al, 1995; Arenberg et al, 1996); however, the function of IL-8 in the other carcinomas has not been clarified. Recently, we found that most gastric carcinomas express IL-8 mRNA and protein, and its level directly correlates with angiogenic activity in the tumour (Kitadai et al, 1998*b*).

In the present study, we performed transfection experiments to obtain direct evidence that IL-8 regulates angiogenesis in gastric carcinoma. IL-8 expression vector was stably transfected into TMK-1 cells (which express negligible levels of IL-8 mRNA and protein) (Kitadai et al, 1998*b*). Transfection with the IL-8 gene did not affect in vitro cell proliferation; however, enforced expression of IL-8 in TMK-1 cells increased their tumorigenic and angiogenic potential in the gastric wall of nude mouse (orthotopic site), thus providing direct evidence for the involvement of IL-8 in angiogenesis.

Although IL-8 transfection increased tumorigenicity and angiogenicity, neither control nor IL-8 transfectants produced distant metastasis. To produce metastasis, tumour cells must complete a series of sequential and selective steps that include growth, vascularization, invasion, adhesion and extravasation (Fidler, 1990). The increase in angiogenic activity by IL-8 transfection may be necessary but not sufficient to produce metastasis by TMK-1 cells.

Angiogenic factors produced by tumour cells and normal cells are critical to the formation of a vascular bed necessary to support tumour growth at the primary and metastatic sites. Because of the complex nature of the angiogenic process, it is unlikely that any one factor is responsible for angiogenesis in a particular tumour type. Within any individual tumour, there may be a dominant angiogenic factor that favours the imbalance of the positive and negative regulators to induce angiogenesis. The two most potent angiogenic molecules are VEGF and bFGF. We previously studied the expression of VEGF and bFGF as well as IL-8 in human gastric carcinoma. The expression level of bFGF is very low and is

*© 1999 Cancer Research Campaign* 

associated with fibrosis in the diffuse type gastric carcinoma (Tanimoto et al, 1991). On the other hand, VEGF is commonly expressed by all the gastric carcinoma cell lines and carcinoma tissues as well as normal mucosa (Yamamoto et al, 1998). Among these angiogenic molecules, IL-8 correlates best with vascularity in the tumours. In addition, we demonstrated that overexpression of IL-8 induced an angiogenic response. Therefore, IL-8 actually regulates angiogenesis in human gastric carcinomas. In this study, we examined the expression of VEGF and bFGF by IL-8 transfectants. There were no changes in the levels of mRNA for VEGF and bFGF after transfection with the IL-8 gene (Figure 3A), indicating that these factors did not play a role in the increased angiogenic activity in this system. Smith et al demonstrated that inhibition of IL-8 using neutralizing antibody resulted in the marked attenuation of angiogenesis in bronchogenic carcinoma (Smith et al, 1994). To prove a possibility for therapeutic intervention for gastric carcinoma, additional studies with neutralizing IL-8 antibody should be required.

IL-8 has been reported to act as an autocrine growth factor for melanoma cells (Schandendorf et al, 1993) and the expression level of IL-8 by human melanoma cells correlates with their metastatic potential (Singh et al, 1994). Furthermore, IL-8 up-regulates collagenase type IV mRNA expression and collagenase activity by melanoma cells (Luca et al, 1997) and stimulate cell motility (Wang et al, 1990). Recent studies have shown that IL-8 receptors, IL-8RA and IL-8RB, are expressed by not only microvessel endothelial cells but also tumour cells in head and neck squamous cell carcinoma (Richards et al, 1997) and breast carcinoma (Miller et al, 1998). Therefore, IL-8 may play an important role in tumour growth and progression by both autocrine and paracrine mechanisms. It would be of great interest to elucidate whether IL-8 receptors are expressed by gastric carcinoma cells.

In conclusion, this study demonstrates that IL-8 produced by gastric carcinoma cells regulates angiogenesis. The identification of factors that correlate with angiogenesis in gastric carcinoma may provide a basis for the design of therapeutic approaches. Studies to determine if attenuation of IL-8 production by gastric carcinomas can produce therapeutic benefits are under way.

### ACKNOWLEDGEMENTS

This work was supported in part by Grants-in-Aid for Cancer Research from the Ministry of Education, Science and Culture of Japan and from the Ministry of Health and Welfare of Japan (YK, ET), grant R35-CA42107 (IJF) from the National Cancer Institute, National Institutes of Health. We thank Walter Pagel for his critical editorial review and Lola Lopez for expert preparation of this manuscript.

### REFERENCES

Arenberg DA, Kunkel SL, Polverini PJ, Glass M, Burdick MD and Strieter RM (1996) Inhibition of interleukin-8 reduces tumorigenesis of human non-small cell lung cancer in SCID mice. *J Clin Invest* **97**: 2792–2802

de Marco D, Kunkel SL, Strieter RM, Basha M and Zurier RB (1991) Interleukin-1-induced gene expression of neutrophil activating protein (interleukin-8) and monocyte chemotactic peptide in human synovial cells. *Biochem Biophys Res Commun* **174**: 411–416

Feinberg AP and Vogelstein B (1983) A technique for radiolabeling DNA restriction endonuclease fragments to high specific activity. *Anal Biochem* **132**: 6–13

Fan D, Bucana CD, O'Brian CA, Zwelling LA, Seid C and Fidler IJ (1990) Enhancement of tumor cell sensitivity to adriamycin by presentation of the drug in phosphyatidylcholine and phosphatidylserine liposomes. *Cancer Res* **50**: 3619–3626

Fidler IJ (1990) Critical factors in the biology of human cancer metastasis: twenty-eighth GHA Clowes Memorial Award Lecture. *Cancer Res* **50**: 6130–6138

Folkman J (1986) How is blood vessel growth regulated in normal and neoplastic tissue? – G.H.A. Clowes Memorial Award Lecture. *Cancer Res* **46**: 467–473

Folkman J (1990) What is the evidence that tumors are angiogenesis-dependent? *J Natl Cancer Inst* **82**: 4–6

Graham CH, Rivers J, Kerbel RS, Stankiewicz KS and White WL (1994) Extent of vascularization as a prognostic indicator in thin (<0.76 mm) malignant melanomas. *Am J Pathol* **145**: 570–574

Green GF, Kitadai Y, Pettaway CA, von Eschenbach AC, Bucana CD and Fidler IJ (1997) Correlation of metastasis-related gene expression with metastatic potential in human prostate carcinoma cells implanted in nude mice using an in situ messenger RNA hybridization technique. *Am J Pathol* **150**: 1571–1582

Gutman M, Singh RK, Xie K, Bucana CD and Fidler IJ (1995) Regulation of interleukin-8 expression in human melanoma cells by the organ environment. *Cancer Res* **55**: 2470–2475

Ito R, Kitadai Y, Kyo E, Yokozaki H, Yasui W, Yamashita U, Nikai H and Tahara E (1993) Interleukin-1α acts as an autocrine growth stimulator for human gastric carcinoma cells. *Cancer Res* **53**: 4102–4106

Ito R, Yasui Y, Kuniyasu H, Yokozaki H and Tahara E (1997) Expression of interleukin-6 and its effect on the cell growth of gastric carcinoma cell lines. *Jpn J Cancer Res* **88**: 953–958

Keane MP, Arenberg DA, Lynch III JP, Whyte RI, Iannettoni MD, Burdick MD, Wilke CA, Morris SB, Glass MC, DiGiovine B, Kunkel SL and Strieter RM (1997) The CXC chemokines, IL-8 and IP-10, regulate angiogenic activity in idiopathic pulmonary fibrosis. *J Immunol* **159**: 1437–1443

Kita M, Ohmoto Y, Hirai Y, Yamaguchi N and Imanishi J (1992) Induction of cytokines in human peripheral blood mononuclear cells by mycoplasmas. *Microbiol Immunol* **36**: 507–516

Kitadai Y, Haruma K, Tokutomi T, Tanaka S, Sumii K, Carvalho, M, Kuwabara M, Yoshida K, Hirai T, Kajiyama G and Tahara E (1998*a*) Significance of vessel count and vascular endothelial growth factor in human esophageal carcinomas. *Clin Cancer Res* **4**: 2195–2200

Kitadai Y, Haruma K, Sumii K, Yamamoto S, Ue T, Yokozaki H, Yasui W, Ohmoto Y, Kajiyama G, Fidler IJ and Tahara E (1998*b*) Expression of interleukin-8 correlates with vascularity in human gastric carcinomas. *Am J Pathol* **152**: 93–100

Koch AE, Polverini PS, Kunkel SL, Harlow LA, DiPietro LA, Elner SG and Strieter RM (1992) Interleukin-8 as a macrophage-derived mediator of angiogenesis. *Science* **258**: 1798–1801

Krueger G, Jorgensen C, Miller C, Schroeder J, Sticherling M and Christopher E (1990) Effect of IL-8 on epidermal proliferation. *J Invest Dermatol* **94**: 545

Luca M, Huang S, Gershenwald JE, Singh RK, Reich R and Bar-Eli (1997) Expression of interleukin-8 by human melanoma cells up-regulates MMP-2 activity and increases tumor growth and metastasis. *Am J Pathol* **151**: 1105–1113

Macchiarini P, Fontanini G, Hardin MJ, Squartini F and Angeletti CA (1992) Relation of neovascularization to metastasis of non-small cell lung cancer. *Lancet* **340**: 145–147

Matsushima K, Morishita K, Yoshimura T, Lavu S, Kobayashi Y, Lew W, Appella E, Kung HF, Leonard EJ and Oppenheim JJ (1988) Molecular cloning of a human monocyte-derived neutrophil chemotactic factor (MDNCF) and the induction of MDNCF mRNA by interleukin-1 and tumor necrosis factor. *J Exp Med* **167**: 1883–1893

Matsushima K, Baldwin ET and Mukaida N (1992) Interleukin-8 and MCAF: novel leukocyte recruitment and activity cytokines. *Chem Immunol* **51**: 236–265

Miller LJ, Kurtzman SH, Wang Y, Anderson KH, Lindquist RR and Kreutzer DL (1998) Expression of interleukin-8 receptors on tumor cells and vascular endothelial cells in human breast cancer tissue. *Anticancer Res* **18**: 77–81

Ochiai A, Yasui W and Tahara E (1985) Growth-promoting effect of gastrin in human gastric carcinoma cell line TMK-1. *Jpn J Cancer Res* **76**: 1064–1071

Radinsky R, Kraemer PM, Raines MA, Kung HJ and Culp LA (1987) Amplification and rearrangement of Kirsten ras oncogene in virus transformed BALB/3T3 cells during malignant tumor progression. *Proc Natl Acad Sci USA* **84**: 5143–5147

Richards BL, Eisma RJ, Spiro JD, Lindquist RL and Kreutzer DL (1997) Coexpression of interleukin-8 receptors in head and neck squamous cell carcinoma. *Am J Surg* **174**: 507–512

Schandendorf D, Moller A, Algermissen B, Worm M, Sticherling M and Czarnetzki BM (1993) IL-8 produced by human malignant melanoma cells in vitro is an essential autocrine growth factor. *J Immunol* **151**: 2667–2675

 © 1999 Cancer Research Campaign

Schroeder J-M and Christopher E (1986) Identification of C5ades arg and an anionic neutrophil-activating peptide (ANAP) in psoriatic scales. *J Invest Dermatol* **87**: 53–58

Singh RK, Gutman M, Radinsky R, Bucana CD and Fidler IJ (1994) Expression of interleukin 8 correlates with the metastatic potential of human melanoma cells in nude mice. *Cancer Res* **54**: 3242–3247

Smith DR, Polverini PJ, Kunkel SL, Orringer MB, Whyte RI, Burdick MD, Wilke CA and Strieter RM (1994) Inhibition of interleukin-8 attenuates angiogenesis in bronchogenic carcinoma. *J Exp Med* **179**: 1409–1415

Smith-MacCune KK and Weidner N (1994) Demonstration and characterization of the angiogenic properties of cervical dysplasia. *Cancer Res* **54**: 800–804

Strieter RM, Kunkel SL, Elner VM, Martonyi CL, Koch AE, Polverini PJ and Elner SG (1992) Interleukin-8: a corneal factor that induces neovascularization. *Am J Pathol* **141**: 1279–1284

Strieter RM, Polverini PJ, Arenberg DA, Walz A, Opdenakker G, Van-Damme J and Kunkel SL (1995) Role of C-X-C chemokines as regulators of angiogenesis in lung cancer. *J Leukoc Biol* **57**: 752–762

Tachibana M, Miyakawa A, Nakashima J, Murai M, Nakamura K, Kubo A and Hata JI (1997) Constitutive production of multiple cytokines and a human chorionic gonadotrophin beta-subunit by a human bladder cancer cell line (KU-19-19): possible demonstration of totipotential differentiation. *Br J Cancer* **76**: 163–174

Tahara E (1990) Growth factors and oncogenes in human gastrointestinal carcinomas. *J Cancer Res Clin Oncol* **116**: 121–131

Tahara E (1993) Molecular mechanism of stomach carcinogenesis. *J Cancer Res Clin Oncol* **119**: 265–272

Takahashi Y, Kitadai Y, Bucana CD, Cleary KR and Ellis LM (1995) Expression of vascular endothelial growth factor and its receptor, KDR, correlates with vascularity, metastasis, and proliferation of human colon cancer. *Cancer Res* **55**: 3964–3968

Takahashi Y, Cleary KR, Mai M, Kitadai Y, Bucana CD and Ellis LM (1996) Significance of vessel count and vascular endothelial growth factor and its receptor (KDR) in intestinal-type gastric cancer. *Clin Cancer Res* **2**: 1679–1684

Tanigawa N, Amaya H, Matsumura M, Shimomatsuya T, Horiuchi T, Muraoka R and Iki M (1996) Extent of tumour vascularization correlates with prognosis and hematogenous metastasis in gastric carcinomas. *Cancer Res* **56**: 2671–2676

Tanimoto H, Yoshida K, Yokozaki H, Yasui W, Nakayama H, Ito H, Ohama K and Tahara E (1991) Expression of basic fibroblast growth factor in human gastric carcinomas. *Virchows Arc B* **61**: 263–267

Van Meir EG, Ceska M, Effenberger F, Walz A, Grouzmann E, Desbaillets I, Frei K, Fontana A and de Tribolet N (1992) Interleukin-8 is produced in neoplastic and infectious disease of the human central nervous system. *Cancer Res* **52**: 4297–4305

Wang JM, Taraboletti G, Matsushima K, Damme JV and Mantovani A (1990) Induction of haptotactic migration of melanoma cells by neutrophil activating protein/IL-8. *Biochem Biophys Res Commun* **169**: 165–170

Weidner N, Semple JP, Welch WR and Folkman J (1991) Tumor angiogenesis and metastasis – correlation in invasive breast carcinoma. *N Engl J Med* **324**: 1–8

Weidner N, Carroll PR, Flax J, Blumenfeld W and Folkman J (1993) Tumor angiogenesis correlates with metastasis in invasive prostate carcinoma. *Am J Pathol* **143**: 401–409

Yamamoto S, Yasui W, Kitadai Y, Yokozaki H, Haruma K, Kajiyama G and Tahara E (1998) Expression of vascular endothelial growth factor in human gastric carcinomas. *Pathol Int* **48**: 499–506

*© 1999 Cancer Research Campaign*
